# Kinetics of Si and Ge nanowires growth through electron beam evaporation

**DOI:** 10.1186/1556-276X-6-162

**Published:** 2011-02-21

**Authors:** Pietro Artoni, Emanuele Francesco Pecora, Alessia Irrera, Francesco Priolo

**Affiliations:** 1 MATIS IMM-CNR, Via Santa Sofia 64, I-95123 Catania, Italy; 2 Dipartimento di Fisica e Astronomia, Università di Catania, Via Santa Sofia 64, I-95123 Catania, Italy; 3 CSFNSM - V.le A. Doria 6, I-95125 Catania, Italy

## Abstract

Si and Ge have the same crystalline structure, and although Si-Au and Ge-Au binary alloys are thermodynamically similar (same phase diagram, with the eutectic temperature of about 360°C), in this study, it is proved that Si and Ge nanowires (NWs) growth by electron beam evaporation occurs in very different temperature ranges and fluence regimes. In particular, it is demonstrated that Ge growth occurs just above the eutectic temperature, while Si NWs growth occurs at temperature higher than the eutectic temperature, at about 450°C. Moreover, Si NWs growth requires a higher evaporated fluence before the NWs become to be visible. These differences arise in the different kinetics behaviors of these systems. The authors investigate the microscopic growth mechanisms elucidating the contribution of the adatoms diffusion as a function of the evaporated atoms direct impingement, demonstrating that adatoms play a key role in physical vapor deposition (PVD) NWs growth. The concept of incubation fluence, which is necessary for an interpretation of NWs growth in PVD growth conditions, is highlighted.

## Introduction

The synthesis and the tailoring of the electrical and optical properties of nanostructured materials are fascinating research fields, and they represent a suitable route in a wide range of potential nanoscale device applications. Among these, axial structures such as C nanotubes and group IV semiconductor nanowires (NWs) are a realistic addition because of the quantum confinement of their carriers in the planar direction and because of their high surface/volume ratio. In the literature many simple device structures have been demonstrated taking advantage of the enhanced electrical properties of the NWs [1-3], of their quantum confinement for light emission [4,5] or detection [6], of the decoupling of the light absorption and carrier extraction for efficient solar cell elements and of the enhanced surface effects as biochemical sensors[7,9], or of their structure for high-performance anode batteries [10]. A broad selection of NW composition and band structures is reported, but group IV semiconductor NWs are the most interesting at the moment because they can be easily integrated with the current CMOS technology. In particular, Si is the leading semiconductor, and its unlimited abundance makes it as the primary element for the future applications. On the other hand, Ge is experiencing a renewed interest, and it has been recently proposed for specific high-frequency applications [11].

Si and Ge NWs can be synthesized following a bottom-up approach, named vapor-liquid-solid (VLS) [12]. By exploiting the self-assembling capability of the semiconductor atoms coming from the vapor phase to diffuse toward metallic droplets to form a eutectic liquid phase and, at the same time, to supersaturate the droplets performing the NWs axial growth, this approach allows the control of all the structural features of the NWs such as length, radius, and crystallographic properties. Gold has been usually chosen as a catalyst, and the influence of its diffusion on the NW sidewall has been extensively investigated [13]. Different techniques usually benefit of the VLS mechanism. Chemical vapor deposition (CVD) has been widely used to grow NWs through the VLS mechanism. The peculiar issue of this technique is the active chemical role of the metal droplet, which catalyzes the cracking of the precursor molecule in such a way that elemental atoms are formed under the gold droplet, and the interaction with the overall substrate is quite absent.

On the contrary, the physical vapor deposition (PVD) techniques involve a different feeding contribution other than direct impingement. In fact, the metal droplet represents a thermodynamic constraint only. It determines the area in which the eutectic conditions are reached. On the other hand, the evaporated Si or Ge atoms reaching the substrate interact with the surface atoms, bond with them, and start to diffuse. They actually act as adatoms, and it is demonstrated that they play a fundamental role in the NWs growth. In particular, the microscopic growth mechanisms governing the Si and Ge NWs growth in electron beam evaporation (EBE) technique are investigated in detail. EBE is a PVD technique and, in contrast to the MBE, it is a non-ultra-high vacuum, very flexible, and economic preparation technique with broad industrial applications due to its very high potential deposition rate. In the very recent times, it has been successfully proposed for the growth of group IV semiconductor NWs because, despite its non-UHV regime, NWs synthesized by EBE have high crystallographic quality (they are single crystal and possibly faceted), and it is possible to control their length, density, as well as their crystallographic growth direction by changing the experimental parameters [14,15].

Si and Ge have the same crystallographic structure, with a lattice misfit of about 4% only. Moreover, the Si/Au and Ge/Au phase diagrams are very similar too: each one has a single eutectic point, placed at substantially the same temperature (about 360°C), and the semiconductor percentages in the alloy at the eutectic temperature are comparable (19 and 28%, respectively) [16]. From a thermodynamic point of view, their behavior with respect to the NW growth by VLS can be considered the same. Nevertheless, in this article, it is demonstrated that Si and Ge NWsgrowth occur in very different temperatures and fluence regimes. The growth mechanisms elucidating the relevance of the kinetic behavior of Si and Ge adatoms on the axial growth rate are investigated in detail. Finally, the contribution of the direct impingement vs the surface diffusing ad-atoms to the NWs growth in a PVD system is clarified.

### Experimental

Samples have been prepared in an EBE chamber which allows multiple subsequent evaporations from dissimilar and separate crucibles. Au pellets, Si ingots, or Ge ingots have been used as the sources. The evaporation flux and the nominal planar film thickness were measured *in situ *through a quartz microbalance. The density of these layers has been measured by comparing the thickness (measured using scanning electron microscope--SEM) with the atomic areal density (measured using Rutherford backscattering spectrometry). In contrast to Si layer, Ge layer grown by EBE shows a deeply terraced surface. Moreover, some voids are visible between terraces, and the effective density of this Ge layer is about a 20% lower than the Ge bulk density. Therefore, the evaporated flux impinging on each sample was set to the value of 2.5 × 10^14 ^cm^-2 ^s^-1 ^in the case of Si and to the value of 1.5 × 10^14 ^cm^-2 ^s^-1 ^in the case of Ge, to obtain the same velocity of growth of the planar films, set at a constant value of 0.05 nm s^-1^. The evaporated fluence has been varied in the range from 0.25 to 2.50 × 10^18 ^atoms cm^-2^. The apparatus is equipped with a substrate holder which can be heated through Joule effect up to 800°C.

(111)-oriented *n*-type Si pieces are used as substrates in all the cases. Sample preparation procedure comprehends surface cleaning (UV oxidation followed by a dip in HF etching) to remove all surface impurities and to avoid any oxygen contamination. In fact, it has been demonstrated that the presence of the native Si oxide inhibits the NWs growth [17,18]. Then, the samples are loaded in the vacuum chamber (base pressure of 1-2 × 10^-8 ^mbar) where a 2-nm-thick Au layer has been first evaporated on top of the sample keeping it at room temperature. After deposition, a thermal annealing at 700°C for 2 h has been conducted to break the continuous layer and induce the formation of gold droplets on the substrate. These steps are repeated for all the samples in such a way that the substrate, the catalyst size distribution, and density are always the same. Then, Si or Ge is evaporated at the desired growth temperature, performing the NWs growth.

Structural characterization is performed using a FE-SEM Zeiss Supra 25. Plan, 65° tilted, and cross images are performed to investigate surface properties, NWs structural features, and layer thicknesses. Statistical analyses are conducted using the Gatan Digital Microscope software. Focused ion beam (FIB) experiments are performed with a 30-keV Ga^+ ^FIB FEI V600.

## Results and discussion

### Growth mechanisms

**Figure 1:**
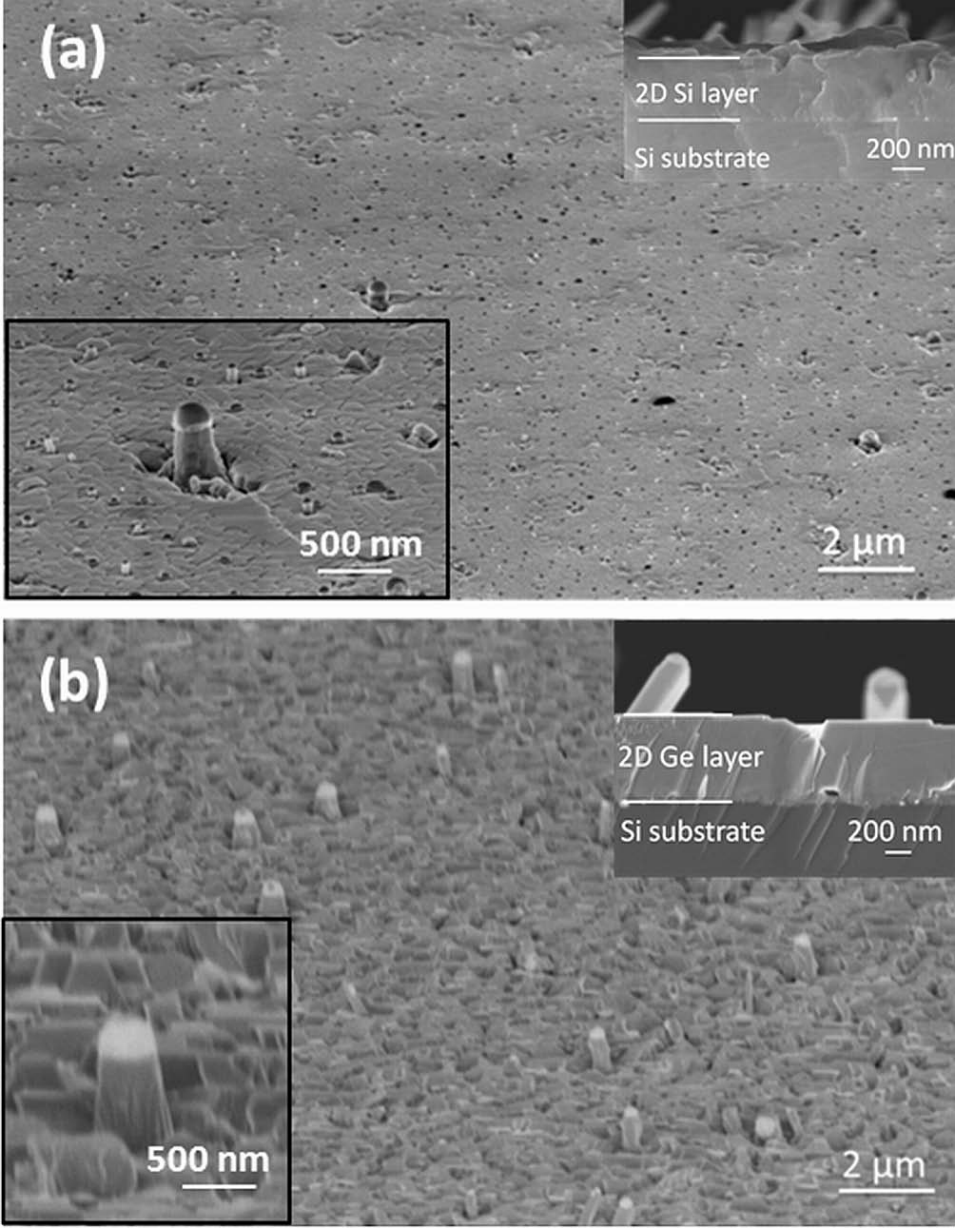
**SEM images of Si NWs and Ge NWs**. **(a) **Low-magnification SEM images of sample of Si NWs. The bottom inset shows a higher magnification of a Si NW. The top inset is a cross-sectional SEM image of the sample showing the substrate and the 2D Si layer on top of it. **(b) **Low-magnification SEM images of Ge NWs. The bottom inset shows a Ge NW. In the top inset, the cross section of the sample is shown, and the Si substrate, the 2D Ge layer, and some NWs are visible

Figure 1 shows the low-magnification SEM images of typical samples of Si (a) and Ge (b) NWs. In particular, these were prepared after evaporation of a Si fluence of 1.75 × 10^18 ^atoms cm^-2 ^(Figure 1a) or a Ge fluence of 1.00 × 10^18 ^atoms cm^-2 ^(Figure 1b). The bottom insets of Figure 1a, b show high-magnification images of Si and Ge NWs samples, respectively. The growth temperature was set at 480°C in both cases. Both Si and Ge NWs are clearly visible with the Au droplet standing on top of them. The growth direction of these NWs is (111) (they are perpendicular to the substrate), since these growth parameters lead to a major percentage of (111) NWs, while other crystallographic directions are observed at different growth temperatures or evaporated fluences, as has already been demonstrated earlier [14,15]. A key issue of the NWs growth by EBE is the competition between the axial growth and the planar growth of a layer all over the sample. In fact, the evaporated atoms reaching the heated substrate from the vapor phase can directly impinge on the gold droplet or interact with the overall substrate, becoming adatoms. Depending on the substrate temperature, they can diffuse on the surface of the sample, and if they are not so far from the Au droplet, then they can diffuse along the NW sidewall eventually reaching the metal/semiconductor interface contributing effectively to the axial growth. On the other hand, the adatoms stop when they form more than one stable bonding with the surface atoms, contributing to the growth of a planar layer. A film is clearly visible both in Si and Ge NWs samples growing on top of the substrate. A cross-sectional SEM images of Si and Ge NWs samples are shown in the top inset of Figure 1a, b, respectively: the Si and Ge layer on top of the Si substrate is visible, and the Si and Ge NWs overcome this layer. Such a competition between the planar versus the axial growth has been modeled by Dubrovskii et al. [19] and it has been observed in the NWs growth both by MBE [20,21] and EBE [14,22,23]. In particular, the presence of a dip around the NWs clearly demonstrates that the atoms missing from the planar layer act as a sort of reservoir contributing to the axial growth of the NWs. The surface area of this dip is named as the "collecting area." Only atoms impinging inside this area can potentially contribute to the NWs axial growth. For an effective contribution, these adatoms should not be desorbed from the substrate, or be adsorbed (in this way, they would contribute to the planar layer growth), and finally they have to be able to reach the growing NW up to the metal/semiconductor interface. The relevant role played by kinetic processes for the NWs growth in PVD techniques is evident as well as the thermodynamic constraints. It has been recently demonstrated for Si grown by EBE, by investigating the role of oxygen contaminations in relation to the adatoms surface diffusivity [18].

In the later sections of this article, the authors will elucidate the adatoms contribution by comparing the Si and Ge growth regimes. In fact, these two semiconductors have strong differences from a kinetic point of view. Despite the presence of adatoms diffusion on the substrate proceeds with the same mechanism (one of the four dangling bonds links with a dangling bond of the surface and diffusion continues till the adatom finds a more stable position where it can saturate two or more dangling bonds), and it is well known that Ge surface diffusivity on Si is very different from the self-diffusion of Si [24]. Moreover, the melting point of Ge is 475°C lower than that of Si, and solid-phase epitaxy regrowth in Ge has a lower activation energy (*E*_Ge _= 2.0 eV) than in Si (*E*_Si _= 2.7 eV), with the same pre-exponential value (about of 3 × 10^8 ^cm s^-1^). As a consequence, recrystallization processes in Ge occur at much lower temperatures with respect to the typical Si temperature processes for crystalline growth [25,26]. The differential bond energy between Si/Si and Ge/Si atoms can account for this difference, and, consequently, for the very different mobilities of these species. Moreover, according to Zakharov et al. [27] referring to the MBE growth technique, atoms directly impinging on the catalyst droplet allow the growth of the NWs in maintaining the Au droplet on top of it with a maximum axial rate that is equal to the planar rate. One could expect that Si or Ge NWs growth is observable in the same regime with similar structural features. On the contrary, it is shown that these two nanostructures grow at different temperatures and different fluence regimes, and these results are correlated to the different Si and Ge adatoms kinetics on the substrate.

### Temperature dependence

**Figure 2:**
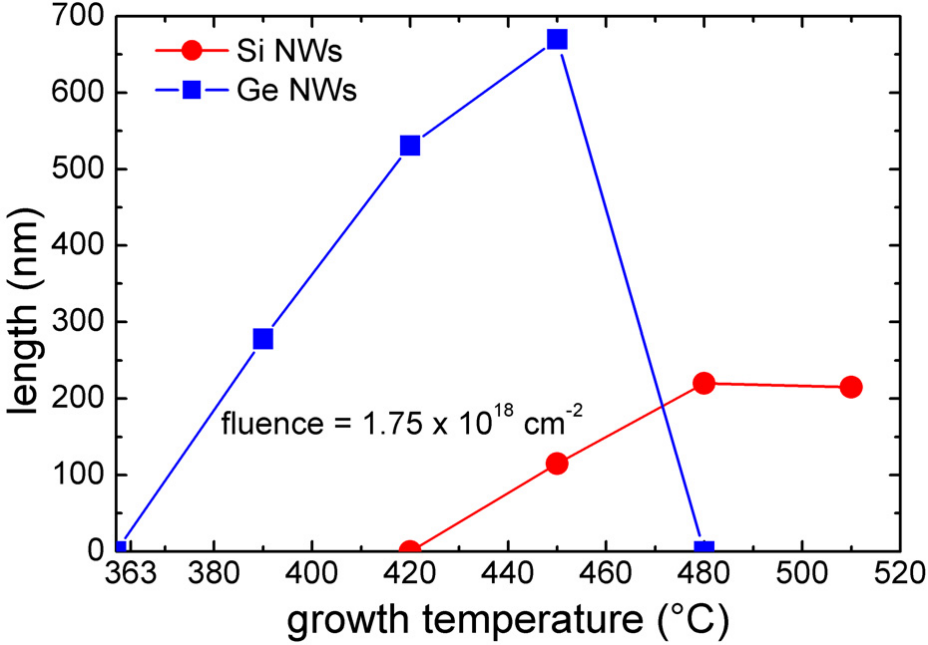
**Si (red dots) and Ge (blue squares) NWs measured length as a function of the growth temperature for an evaporated fluence of 1.75 × 10^18 ^cm^-2^**.

Figure 2 reports the Si (red dots) and Ge (blue squares) NWs lengths as a function of the growth temperature for an evaporated fluence of 1.75 × 10^18 ^cm^-2^. The measured NWs length increases as the temperature increases up to a maximum value which is obtained at 450°C for the Ge and 480°C for the Si NWs. At higher temperatures, the length saturates and, as is well evident for Ge NWs, it decreases, and NWs growth is eventually inhibited. This trend resembles a bell-shaped behavior, with the length reaching its maximum value at an intermediate temperature. This is the result of the competition between two different and opposite temperature-dependent processes, both related to the adatoms contribution to the axial growth. The first one is the adatoms diffusion which is brought about by increasing the substrate temperature. As a consequence, the adatoms surface diffusivity increases, and the collecting area enlarges. The axial NWs growth increases because of the increased number of the contributing adatoms. Instead, adatoms can desorb from the substrate and come back into the gaseous phase; the rate of this process is increased by further increasing the substrate temperature, making it detrimental for the growth.

It is intriguing to note that Si and Ge NWs growth occurs in very different regimes of temperature. In fact, Ge NWs grow, which can be observed just above the eutectic temperature (363°C). On the other hand, the minimum temperature at which Si NWs are observed is 450°C. The authors performed specific experiments at lower temperatures (360 and 420°C, respectively), but no NWs were observed in the samples. The existence of a lower bound temperature which is well above the eutectic temperature is not generally observed in some growth techniques, such as CVD growth. In fact, in the CVD technique, the semiconductor (Si or Ge) adatoms diffusion on the surface plays a minimal role with respect to direct impingement of the semiconductor gaseous species on the metallic droplet. Indeed, in PVD case it is concluded that, because of the different Si and Ge surface diffusivity, Si NWs growth needs a temperature very much higher than the Au-Si eutectic temperature, whereas Ge NWs growth is essentially limited by the eutectic temperature in such a way that thermodynamics sets a lower bound condition.

Finally, another difference arises because of the NWs length itself; while Si NWs at these conditions reach a maximum length of 200 nm, Ge NWs are taller by about a factor of 4. This evidence is strictly related to the differential axial rate behavior with respect to the temperature and the evaporated fluence of the two semiconductors; the dependence due to the latter will be discussed in the next section.

### Competition between axial and 2D growth rates

**Figure 3:**
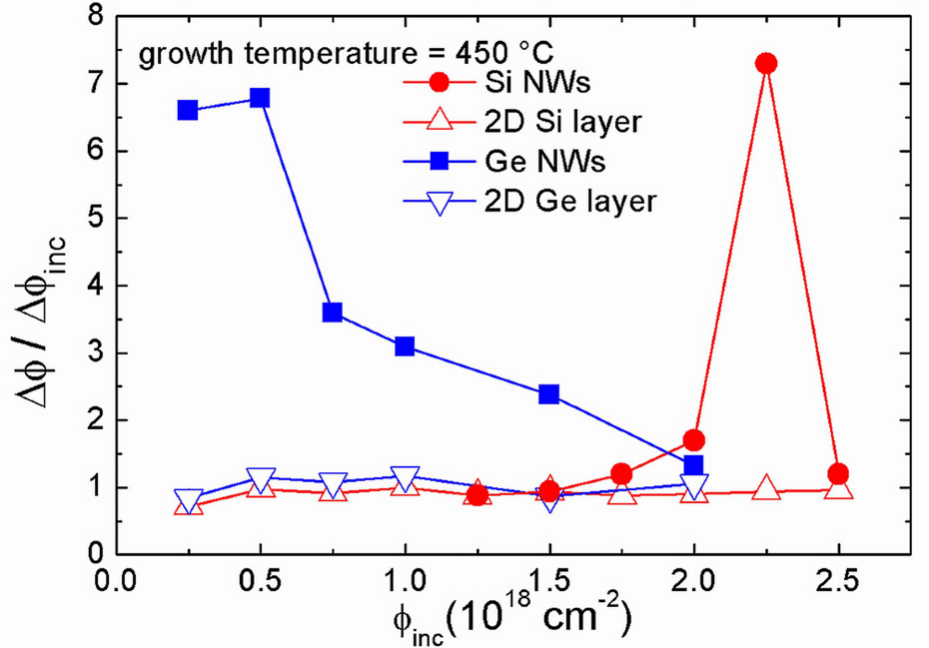
**Increment of the fluence ΔΦ of both the NWs, and the planar rate over the increment of evaporated incident fluence ΔΦ_inc_, as a function of the evaporated fluence Φ_inc_**. In particular, in the case of the NW, ΔΦ_NW _has been calculated as the increment of the areal densities of atoms contributing to the NW growth. This ratio represents the axial growth rate of the NW derived with respect to the evaporated fluence.

A comprehensive comparison of the axial growth rate in the case of Si and Ge NWs synthesized by EBE is shown in Figure 3. This figure reports the increment of the fluence ΔΦ of both the NWs and the planar rate over the increment of the evaporated incident fluence (ΔΦ_inc_), as a function of the evaporated fluence Φ_inc_. In particular, in the case of the NW, ΔΦ_NW _has been calculated as the increment of the areal density of atoms contributing to the NWs growth. This ratio represents the axial growth rate of the NW derived with respect to the evaporated fluence.

Red dots and blue squares refer to the NW contributions of Si and Ge NWs, respectively. In both cases, the growth temperature of 450°C and (111)-oriented NWs only are taken into consideration, which in these growth conditions are the most observed directions. Red and blue triangles refer to the planar rate of Si and Ge layers, respectively. These values have been obtained from the cross SEM measurements of the thicknesses of the planar layers grown by evaporation, and the duration of the evaporation, by considering the different densities of Si and Ge 2D layers grown by EBE.

Differences between Si and Ge are very impressive. In fact, the axial rate of Si NWs increases only at high evaporated fluences. The minimum Si fluence necessary to observe Si NWs outside the planar layer is equal to 1.75 × 10^18 ^cm^-2^. This value is defined as the incubation fluence for the growth. Moreover, after the conditions for the catalyzed growth are reached, the axial growth occurs in a limited range of evaporated fluences (from 1.75 to 2.50 × 10^18 ^cm^-2^), but it is very efficient being about seven times higher than the planar rate. At the fluence value of 2.50 × 10^18 ^cm^-2^, it assumes again the planar rate value. On the other hand, the behavior of Ge is very different. The incubation fluence is strongly reduced, being less than 0.25 × 10^18 ^cm^-2^, i.e., the growth after evaporating a small Ge fluence is observed, which is equivalent to a planar layer of about a few nanometers. The axial rate of Ge NWs is first about seven times higher than the planar rate, and then it continuously decreases on increasing the evaporated fluence until it comes back to the planar value. The fact that the peak values of the axial rates in both Si and Ge NWs are quite similar can be attributed to the similar mechanism of surface diffusion of Si and Ge adatoms.

### Direct impingement versus adatoms contribution

**Figure 4:**
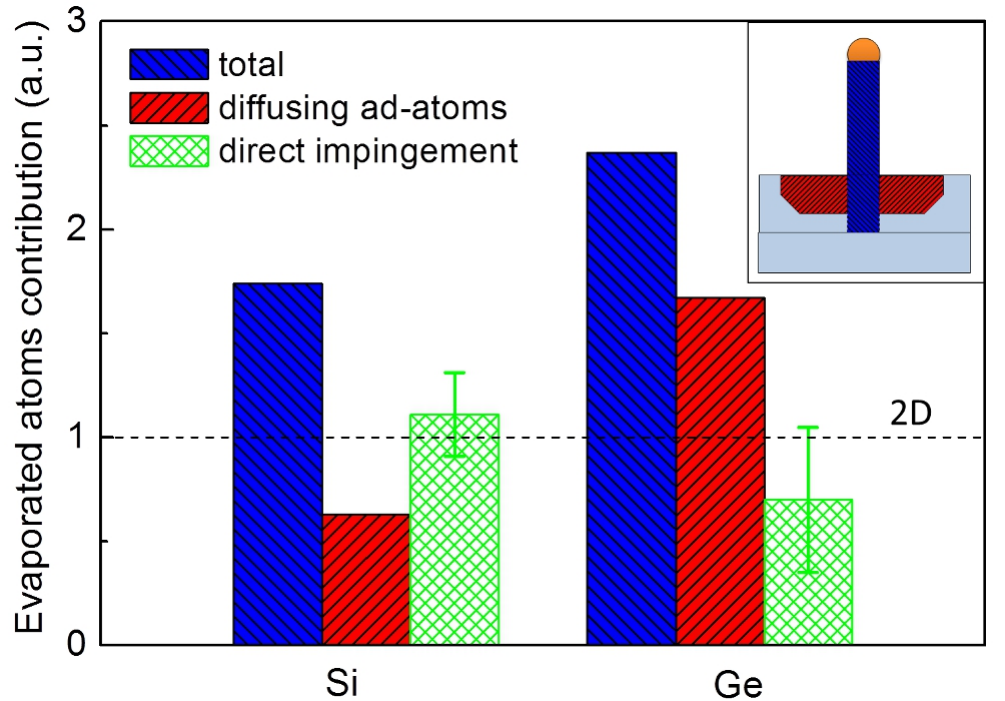
**Measured volume of the entire NW (blue column); measured volume due to the contribution of the Si or Ge diffusing adatoms (red columns); difference between the overall volume and the part ascribed to the adatoms (green columns)**. The calculated volume *V*_2D _which should be filled by a completely planar layer after an evaporation of such a fluence is reported in the graph with the dashed line. All data are normalized to this value.

It is demonstrated that surface adatoms diffusion has a relevant role on the NWs growth, determining the collecting area and consequently the axial growth rate. Temperature and evaporated fluence dependences support this model. On the other hand, in the typical description of the VLS mechanism, the main role is ascribed to the atoms impinging on the Au droplet, then to those diffusing into it and reaching the liquid interface. In order to quantify, which is the effective role of the two processes (direct impingement vs adatoms diffusion form the surface) in the PVD techniques, both in the cases of Si and Ge evaporations, a specific experiment that can evaluate the volume of the dip around the NWs is performed. The dip is a sort of reservoir such that the atoms missing in this volume have been consumed for the NWs growth, thus contributing to its total volume. In particular, through FIB cross sections of single Si (and Ge) NWs were locally performed, both of them being prepared at a growth temperature of 480°C; the evaporated fluence has been chosen such that the thickness of the planar layer is constant. In particular, half of the NW and the surrounding grown layer were vertically cut till the Si wafer substrate to make visible a section of the dip around the NW. The volume of this dip was measured, corresponding to the evaporated adatoms contribution to the axial growth. Furthermore, the entire volume of the NWs was measured. Since the densities of Si and Ge are different, and since the measured NWs have different radius, data are analyzed to make direct comparison possible. Both the NW and the dip volumes to the volume of a cylinder having the same radius of the NW and the same height of the 2D planar layer, named *V*_2D_, were normalized. In this study, the total and the adatoms contributions to the NW growth were obtained, which are reported in Figure 4 with blue and red columns, respectively, for both Si and Ge. In the inset of the figure, a schematic picture of the experiment is depicted. A section of the NW is drawn, and the measured volumes (of the dip and of the NW) are colored according to the column in the graph. To complete the description, it is necessary to quantitatively evaluate the contribution of the atoms which directly impinge on the Au droplet and are adsorbed into the liquid interface through the catalyst. With this purpose in view, the difference between the total NW volume and the volume of the dip was calculated. The properly normalized difference is reported in the green columns, and it represents the direct impingement contribution. The height of the green column has to be compared with the volume *V*_2D _which should be filled by a completely planar layer after an evaporation of such a fluence. This volume refers to a 2D planar layer grown under the same conditions without the presence of the gold droplet. This calculated value is reported in the graph with the dashed line.

It is remarkable to observe that the volume ascribed to the direct impingement process on the NW growth matches very well with the volume *V*_2D _which should be filled by the planar layer in the absence of the Au droplet. In other words, this analysis definitely demonstrates that direct impingement, in the case of PVD techniques, has a minor role in the axial growth because it contributes to a maximum NW height corresponding to the thickness of the planar layer only. NWs should not be visible outside the planar layer if direct impingement were the only mechanism for the axial growth. On the contrary, it is demonstrated that adatoms diffusion has a relevant role in the axial growth. The measured length outside the 2D film is due to this mechanism only.

## Discussion

On the basis of the data reported in this article, the authors have been able to model the NWs growth by PVD techniques. In particular, the differences between Si and Ge NWs behaviors will drive this modeling. In this case, the substrates are always Si wafers. When Si is evaporated, Si adatoms diffusion on Si during the whole growth process must be taken into consideration. On the contrary, at the first stages of Ge evaporation, Ge adatoms move on Si. Later, the Si from the substrate cannot interact anymore with the Ge adatoms, and they start to move on a Ge planar layer. It is reported in the literature that the diffusion mean length measured at 450°C of Ge on Si is twice greater than that of Si on Si [24]. Moreover, the diffusion mean length of Ge on Ge is about a factor of 15 times higher than that of Si on Si. As a consequence, by changing the mean diffusion length in the different systems, the effective collecting area for the growth is changed. In particular, the collecting area for Ge NWs is much greater than for Si NWs. As a consequence, once the substrate temperature is fixed, the incubation fluence value for Ge NWs growth can be reached at lower fluence values with respect to those of the Si.

**Figure 5:**
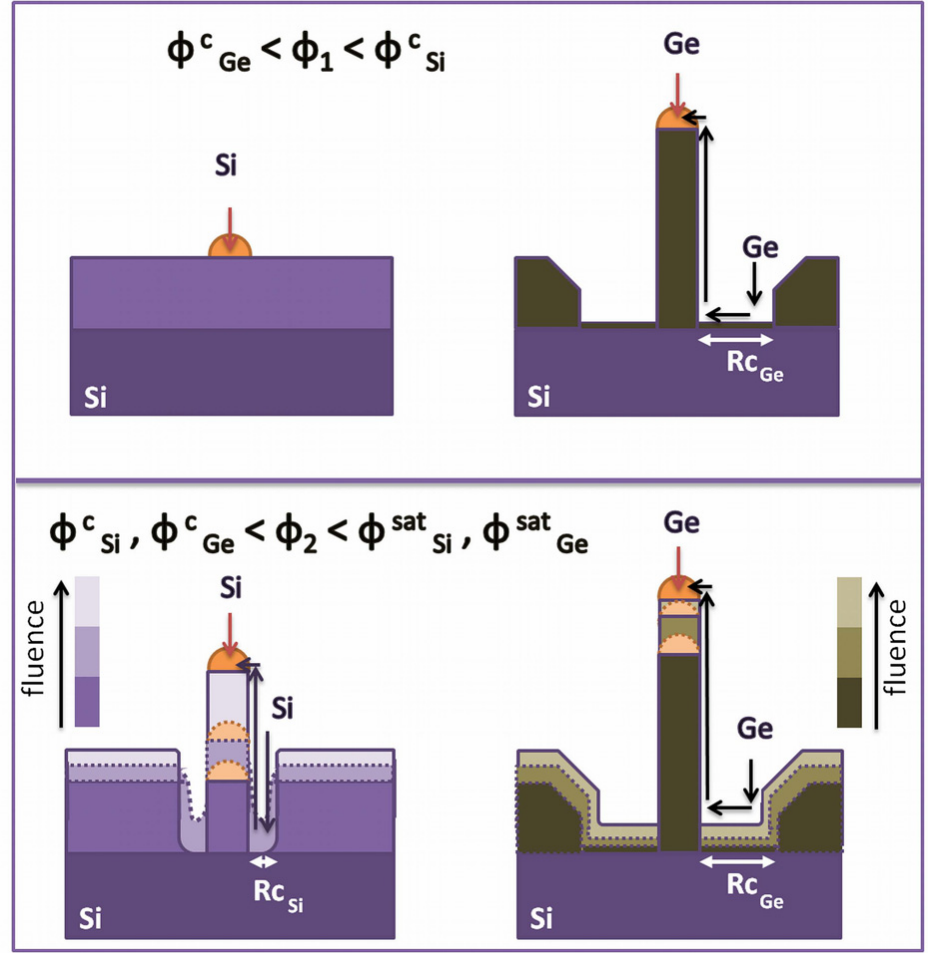
**Schematic picture of the Si NWs (left-hand side) and of the Ge NWs growth on Si substrate (right-hand side), in different fluence regimes**. Color scale refers to the evolution of the growth as a function of the evaporated fluence.

Figure 5 shows the schematic picture of the Si (left-hand side) and of the Ge NWs (right-hand side) growth on a Si substrate. Color scale refers to the evolution of the growth as a function of the evaporated fluence, as indicated in the scale bar. The top panel refers to the first stages of the growth, corresponding to an evaporated fluence, named Φ_1_, at which Si NWs are still not observable outside the planar layer, while Ge NWs have started to grow with their maximum possible axial rate. In other words, Φ_1 _is higher than the Ge incubation fluence Φ^c^_Ge _and less than the Si incubation fluence Φ^c^_Si_, i.e., in the range between 0.25 and 1.75 × 10^18 ^cm^-2^. It is clear that Si axial rate is equal to the planar one, but the gold droplets are still active as they have not been covered and they are visible from the top of the sample. On the other hand, Ge adatoms are contributing to the planar layer also, but as they can move on the surface faster than Si adatoms, the Ge incubation fluence has been reached, and we observe very tall Ge NWs despite the low evaporated fluence, and the dip around the NW just being formed. The picture represents this stage. The Ge adatoms path from the dip to the liquid eutectic interface is indicated by arrows. The width of the dip is correlated to the Ge adatoms mean diffusion length, Rc_Ge_. The bottom panel refers to the subsequent stages, in which both Si and Ge NWs are growing. This occurs at evaporated fluences higher than the Si and Ge incubation fluences but less than the respective saturation fluences, named, Φ^sat^_Si _and Φ^sat^_Ge_. Strong differences are observable. In fact, the picture clearly depicts what we discussed about the growth rate measurements in Figure 3. Si NWs are growing with an axial rate which increases with increasing evaporated fluence (note the color scale in the picture) so that the Si NWs length strongly increases at the later stages only. Actually, the total Si NWs length is lower than that of Ge NWs. The dip in this case is also visible, and it is continuously used as a reservoir for the growth. Its width, being determined by the Si adatoms diffusion length Rc_Si_, is narrower than that of Ge. In the fluence regime that are now being analyzed, the Ge axial growth rate is decreasing with increasing evaporated fluence. In fact, the Ge NW is so tall that Ge adatoms cannot reach the gold droplet, because of their finite diffusion length. Therefore, the contribution of the adatoms for the growth is reduced, and adatoms are favored to contribute to the planar layer growth. As a consequence, the Ge NWs length measured outside the planar layer saturates. At the final stage, the collecting area has been totally filled by the adatoms. If the diffusion mean length could be similar for Si and Ge, then NWs should grow in the same regime. Actually, this condition requires either a Ge growth temperature less than the eutectic one or a Si growth temperature so high that desorption process would be dominant.

## Conclusions

This study highlights the microscopic mechanisms occurring during the growth of Si and Ge NWs. It is demonstrated that they grow in different regimes of temperatures and fluences, despite Si and Ge having the same structure, and despite Si-Au and Ge-Au phase diagrams being very similar. First, it was proved that the minimal Si NWs growth temperature is limited by kinetics constrains. From a thermodynamic point of view, the growth could occur above 363°C. Owing to the low activation energy of the surface Si diffusion process, at temperatures less than 420°C, adatoms cannot contribute to the growth. They are substantially frozen on the substrate (i.e., their mean diffusion length is very short), and they cannot contribute to the axial growth. As a consequence, NWs are not visible outside the planar layer. On the contrary, the minimal Ge NWs growth temperature is limited by the thermodynamic constraint only (the eutectic temperature). Moreover, incubation fluences have been identified for both Si and Ge, and this value is shwon to be much higher in Si NWs than in Ge ones. Accordingly, Si NWs can grow in a very narrow fluence range at higher values than the Ge NWs. We showed that the different Si and Ge surface kinetics can well explain these differences, and we are able to model the microscopic growth mechanisms of both systems. These results open the way for an understanding of the peculiarity of the VLS mechanism in PVD systems, such as EBE to easily control the NWs growth mechanisms in achieving the maximum possible axial rate for both systems.

## Abbreviations

CVDP: chemical vapor deposition; EBE: electron beam evaporation; FIB: focused ion beam; NWs: nanowires; PVD: physical vapor deposition; VLS: vapor-liquid-solid.

## Competing interests

The authors declare that they have no competing interests.

## Authors' contributions

PA participated in the realization of the project, he carried out the experiments and wrote the paper. EFP participated in the realization of the project, in the experiments and in the writing of the paper. AI participated in the realization of the project, she supervised the experiments and the writing of the paper. FP supervised the whole project, the experiments and the interpretation.
